# Endothelial-to-mesenchymal transition in anticancer therapy and normal tissue damage

**DOI:** 10.1038/s12276-020-0439-4

**Published:** 2020-05-28

**Authors:** Kyu Jin Choi, Jae-Kyung Nam, Ji-Hee Kim, Seo-Hyun Choi, Yoon-Jin Lee

**Affiliations:** 1 Division of Radiation Biomedical Research, Korea Institute of Radiological & Medical Sciences, Seoul, 139-706 Korea; 2 Department of Surgery, Memorial Sloan Kettering Cancer Center, New York, NY 10065 USA

**Keywords:** Cancer microenvironment, Cancer microenvironment

## Abstract

Endothelial-to-mesenchymal transition (EndMT) involves the phenotypic conversion of endothelial-to-mesenchymal cells, and was first discovered in association with embryonic heart development. EndMT can regulate various processes, such as tissue fibrosis and cancer. Recent findings have shown that EndMT is related to resistance to cancer therapy, such as chemotherapy, antiangiogenic therapy, and radiation therapy. Based on the known effects of EndMT on the cardiac toxicity of anticancer therapy and tissue damage of radiation therapy, we propose that EndMT can be targeted as a strategy for overcoming tumor resistance while reducing complications, such as tissue damage. In this review, we discuss EndMT and its roles in damaging cardiac and lung tissues, as well as EndMT-related effects on tumor vasculature and resistance in anticancer therapy. Modulating EndMT in radioresistant tumors and radiation-induced tissue fibrosis can especially increase the efficacy of radiation therapy. In addition, we review the role of hypoxia and reactive oxygen species as the main stimulating factors of tissue damage due to vascular damage and EndMT. We consider drugs that may be clinically useful for regulating EndMT in various diseases. Finally, we argue the importance of EndMT as a therapeutic target in anticancer therapy for reducing tissue damage.

## Cell biology: effect of changes in cell type on disease

A process of cellular conversion known as endothelial-to-mesenchymal transition (EndMT) may offer a valuable target for treating cancer and other diseases. In EndMT, the cells lining blood vessels undergo a striking change in shape and physiology, acquiring features of cells called fibroblasts. Fibroblasts form the body’s connective tissue, but also produce scar tissue that impairs organ function. Researchers led by Yoon-Jin Lee of the Korea Institute of Radiological & Medical Sciences in Seoul, South Korea, have reviewed the impact of this transformation on human disease. EndMT is seen as a prelude to heart failure, in lung tissue affected by pulmonary fibrosis, and within tumors, where the process recruits cells that further stimulate cancer progression. The authors highlight the potential of using drugs that target EndMT to bolster the efficacy and safety of tumor therapy.

## Introduction

Endothelial-to-mesenchymal transition (EndMT) was initially observed by electron microscopy in 1975 during a detailed analysis of endocardium differentiation during the formation of heart valves in vertebrate embryogenesis^1^. This process has also been observed in pathological contexts, such as vascular calcification^2^, atherosclerosis^3^, and cardiac and pulmonary fibrosis^4^. Endothelial cells (ECs) display high plasticity, particularly under pathological conditions and in EndMT. During EndMT, endothelial cells lose their characteristic markers, such as CD31, VE cadherin, Tie2, and vWF, while gaining increased expression of mesenchymal markers, such as fibroblast-specific protein-1 (FSP1), alpha 2 smooth muscle actin (α-SMA), and type I/III collagen. Transforming growth factor (TGF)-β1 may induce a phenotypic transformation of proliferating endothelial cells into fibroblast-like cells. TGF-β1, TGF-β2, and bone morphogenetic proteins are also well-known to cause EndMT^5,6^. Several groups have reported on signaling systems and molecules that induce EndMT, including Wnt/β-catenin^7^ and Notch signaling^8^, as well as hypoxia^9^ and oxidative stress^10^. These signaling pathways are related to transcription factors involved in mesenchymal transition, such as Snail, Slug, ZEB1, and ZEB2^11,12^. EndMT exhibits phenotypic changes similar to the epithelial–mesenchymal transition (EMT), which is considered the main process driving phenotypic changes of cells into mesenchymal cells. TGF-β also plays important roles in the EMT process in the tumor microenvironment during tumorigenesis and metastases, and in fibroblastic changes of epithelial cells that occur during the development of fibrotic diseases. EndMT has been further studied in atherosclerosis and tumor angiogenesis^13,14^. Recently, increasing evidence has suggested that EndMT-related processes are important contributors to the microenvironmental plasticity of cancerous tumors^4^. EndMT leads to the production of cancer-associated fibroblasts (CAFs)^15^, which affect tumor growth and metastases. ECs can differentiate via EndMT into adipocytes and mural cells, such as pericytes and smooth muscle cells^16^. It has also been reported that endothelial progenitor cells differentiate into smooth muscle-like progenitor cells via TGF-β1-driven EndMT^17^. Moreover, tumor EndMT induces the formation of both a CAF-associated tumor microenvironment and aberrant tumor vessels^18,19^. Here, we review the role of EndMT in tissue fibrosis and cancer. We focus on EndMT in lung and cardiac tissue, which are often injured during anticancer therapy. In addition, we discuss the therapeutic advantages of targeting EndMT in anticancer therapy.

## EndMT effects on tissue damage

### EndMT in cardiac tissue damage

Cardiac fibrosis is a common feature in patients with advanced heart failure. Anti-fibrotic therapy may be useful for improving the function of a diseased heart, but the development of therapies has been limited by the incomplete understanding of the origin of fibroblasts in the heart. Cardiac fibrosis occurs because of excessive deposition of the extracellular matrix, which is mediated by the reduction of microcirculation and destruction of the normal myocardial structure. Cardiac fibrosis is associated with the appearance of EndMT^20^. TGF-β1 caused EndMT in Tie1Cre;R26RstoplacZ mice (in which the endothelial origin is marked by lacZ expression) and FSP1-GFP mice (expressing green flourescent protein (GFP) under the promoter activity of FSP1), whereas bone morphogenetic protein-7 preserved the endothelial phenotype and inhibited cardiac fibrosis in mice with pressure overload and chronic allograft rejection (Table 1)^20^. EndMT also occurs during the neohypertensive reaction following grafting of veins into the arterial circulation in both mice and humans. An intervening vein graft was evaluated in different mouse models, including a constitutive Endotrack^YFP^ model and an Endotrack^LacZ^ and tamoxifen-induced Endotrack^YFP^ transgenic mouse model. Staining for endothelial-specific markers revealed that in neointimal (but not luminal) YFP^+^ cells, the expression of endothelial-specific markers was lost after grafting. In addition, the expression of the vascular smooth muscle cell markers, SMA and smooth muscle protein 22-alpha (SM22a), progressively increased in YFP^+^ cells (Table 1). Previous data revealed TGF-β–Smad2/3–Slug signaling as a principal pathway regulating EndMT during vein graft remodeling^21^. A study using ET-1^f/f^;Tie2-Cre transgenic mice with specific ET-1 inhibition showed that diabetes mellitus-induced cardiac fibrosis was associated with the emergence of fibroblasts, and that ET-1 further promoted cardiac fibrosis and heart failure through the accumulation of fibroblasts via EndMT (Table 1)^22^. It has been reported that hypoxic conditions can induce EndMT in cardiac damage. Hypoxia induced EndMT in human coronary endothelial cells, an effect mediated by hypoxia-inducible factor (HIF)-1α-induced activation of SNAIL, an EndMT master-regulatory transcriptional factor^23^.

**Table 1:** The effects of miRNAs on EndMT.

miRNA	Target	Function	Research model	Refs.
miR-200b	TGF-β-dependent Smad2/Snail1	Inhibit EndMT	Retina in diabetic animals	^108^
miR-18a-5p	Notch2	Attenuate EndMT	Diabetic cardiomyopathy	^110^
miR-126a-5p	TGF-β signaling	Increase EndMT	Neonatal pulmonary hypertension	^49^
miR-302c	Metadherin	Inhibit EndMT	Hepatocellular carcinoma	^53^
miR-199a-5p	Snail/miR-199a-5p axis	Promote EndMT via the Snail/miR-199a-5p axis	Irradiated human umbilical vein endothelial cells	^95^

### EndMT in lung injury

Recently, EndMT was reported to play a role in pulmonary fibrosis. Chronic hypoxia is an important contributing factor to pulmonary hypertension. EndMT was observed in the pulmonary arteries of rat models of pulmonary hypertension. In pulmonary microvascular ECs, hypoxia-induced HIF‑1α activity modulates the transdifferentiation of ECs via Twist^24^. It has been reported that vildagliptin (a dipeptidyl peptidase 4 [DPP-4] inhibitor that serves as an antidiabetic drug) improves vascular dysfunction. In a model of lipopolysaccharide-induced septic lung injury, vildagliptin reduced pulmonary fibrosis by attenuating EndMT (Table 1)^25^. Recent data demonstrated that the mesenchymal transition of pulmonary capillary ECs served as the origin of fibroblasts found during bleomycin-induced pulmonary fibrosis using the Tie2-Cre/CAG-CAT-LacZ double-transgenic mouse system, which enabled tracing of LacZ expression in Tie2-positive ECs^26^. Cotreatment with TGF-β and activated Ras caused a persistent EndMT phenotype, inducing α-SMA expression, which was not induced by TGF-β or activated Ras alone. The data showed that, via TGF-β signaling, Ras/MAPK regulated the completion of EndMT in bleomycin-induced pulmonary fibrosis^27^. Nintedanib is a tyrosine kinase inhibitor that targets platelet-derived growth factor (PDGF) and the fibroblast growth factor receptor, and reduces vascular remodeling-related neointimal lesions and the medial wall thickness of pulmonary arteries. Moreover, Twist expression and related EndMT were reduced by treatment with nintedanib^28^.

## Role of oxidative stress and hypoxia in EndMT

Reactive oxygen species (ROS) have been reported to induce EndMT during oxidative stress^10,29^. Oxidative stress promotes TGF-β1 secretion, a key inducer of EndMT^30^. Oxidants can directly activate latent TGF-β^31^. In addition, many oxidants can activate the nuclear factor-kappa B (NF-κB) pathway^32^, and subsequently induce the production of inflammatory cytokines, which directly stimulate EndMT^33,34^.

Recent reports have shown that EndMT contributes to fibroblast accumulation during heart and kidney fibrosis and cancer^6,13,35^. Although ROS, TGF-β, inflammatory cytokines, and hypoxia are known to be involved in the development of chronic fibrosis in the lungs, the precise mechanism underlying the progression of initial radiation injuries to chronic fibrosis remains unclear^36,37^. It has also been demonstrated that radiation-induced hypoxia can trigger EndMT through HIF-1α-mediated activation of TGF-β1/Smad signaling in human pulmonary artery ECs. Moreover, regulating EndMT has been suggested as an effective strategy for stopping radiation-induced pulmonary fibrosis at an early stage^38^. Cells derived from EndMT were previously found during atherosclerotic development in intimal plaques, and this process was guided by TGF-β signals, oxidative stress, and hypoxia in vitro and in vivo in mice^39^.

Salvianolic acid A (which has been widely used for the clinical therapy of various diseases involving vascular disturbance) upregulates CD31 and downregulates α-SMA, and can attenuate EndMT, suppresses oxidative stress, and alleviates pulmonary vascular remodeling. The Nrf2/HO-1 signaling pathway may be involved in these processes^40^.

The inhibition of ROS production in ECs reduces oxidative stress-induced EndMT. Brain and muscle ARNT-like protein-1 (BMAL1), an essential clock transcription activator, can prevent the accumulation of intracellular ROS caused by lipoproteins, and subsequent EndMT in human endothelial aortic cells^41–43^ in vitro. In addition, BMAL1 deficiency aggravated intracellular ROS accumulation and EndMT progression via bone morphogenetic protein-mediated signaling. These results provide a mechanism to explain the central role of BMAL1 in atherosclerosis progression, and the phenotype switch of plaque cells^43^. Low-intensity pulsed ultrasound protects endothelial human aortic cells from oxidative stress-induced EndMT, which has been associated with PI3K/AKT signaling^44^. As previously reported, EndMT occurs when oxidative stress and NF-κB activity are increased in ECs by TGF-β1 and TGF-β2^45,46^. Taken together, these findings suggest that inflammation, hypoxia, and endothelial oxidative stress exacerbate EndMT by inducing canonical TGF-β signaling^30^. NADPH oxidases and ROS play key roles in mediating fibrotic responses induced by TGF-β via Smad2/Smad3 activation^30^. Oxidative stress can induce EndMT conversion via TGF-β1- and TGF-β2-dependent pathways and the intracellular ALK5/Smad3/NF-κB pathway^30,47^.

Recent findings have also suggested that EndMT can be regulated using nanoparticles or natural compounds. Polyglucose sorbitol carboxymethyether-modified Fe_2_O_3_ (PSC-Fe_2_O_3_) iron oxide nanoparticles decreased the expression of the endothelial markers CD31 and VE cadherin at an acutely noncytotoxic concentration, and increased the expression of the mesenchymal marker α-SMA. FSP, via its peroxidase-like activity, enhanced EC migration and inhibited angiogenic function, which clearly indicated the occurrence of EndMT in ECs^30^. EndMT leads to vascular damage during angiotensin-II-treated chronic inflammation, which can be prevented by treatment with schisandrin B. Schisandrin B is a natural product derived from *Schisandra chinensis* that can inhibit the activation of NF-κB. Schisandrin B was also found to suppress inflammation/ROS-mediated EndMT by inhibiting NF-κB^48^. Hypoxia is the main factor promoting the occurrence of EndMT. The relationship between hypoxia and TGF-β signaling is regulated by the expression of microRNAs (miRNAs). miR-126a-5p, which inhibits TGF-β signaling, was upregulated in hypoxia-induced persistent pulmonary hypertension of newborns as a cardiac syndrome (Table 2)^49^. Chronic hypoxia increased oxygen consumption and activated fibroblasts in cardiac fibrosis, resulting in aberrant ventricular remodeling^50^. Under hypoxic conditions, the EndMT of human cardiac microvascular ECs promoted tube formation. Autophagy provides protective effects against the EndMT of human cardiac microvascular ECs by degrading Snail under hypoxic conditions^51^. In addition, it has been suggested that hypoxia induces EndMT in human coronary ECs via Hif1a-activated Snail, indicating that endocardium-derived ECs undergo EndMT^23^.

**Table 2:** Genetically engineered mouse models (GEMMs) used to study EndMT.

GEMM(s)	Genetic change(s)	Biological effects	Research animal model	Refs.
Tie1Cre;R26RstoplacZ and FSP1-GFP mice	EC-specific LacZ expression and fibroblast-specific GFP expression	The phenotypic change in ECs was traced in cardiac fibrosis	Cardiac fibrosis	^20^
Tamoxifen-induced Endotrack^YFP^	EC-specific YFP expression	YFP^+^ cells increased SMA and SM22a expression after vein grafting	Vein-grafting models	^21^
ET-1^f/f^;Tie2-Cre	EC-specific ET-1 deletion	ET-1 deletion enhanced fibroblast accumulation	Diabetes mellitus-induced cardiac fibrosis	^22^
Tie2-cre;R26Rosa-lox-Stop-lox-LacZ	EC-specific LacZ expression	Fibroblastic tumor cells were derived from EndMT	B16F10 tumor	^52^
Tie2-Cre Met^fl/fl^	EC-specific Met deletion	Met inhibited GBM-associated fibroblast-like cells with an EC origin	GBM	^19^
RIP1-Tag2;Eng^+/−^	Endoglin-deficient tumor	Endoglin-deficient tumors exhibited the hallmark of EndMT	Pancreatic neuroendocrine tumor	^77^
Tie2-p53^fl/fl^	EC-specific p53 deletion	p53 deletion inhibited EndMT in the tumor vasculature	Radiation therapy for lung cancer	^18^
Tie2-TGFbR2^fl/fl^	EC-specific TGFbR2 deletion	TGFbR2 deletion increased radiation-induced tumor EndMT	Radiation therapy for lung cancer	^18^
Hey2^flx/flx^/Ve-CadCre^−/−^	EC-specific Hey2 deletion	Hey2 deletion decreased the EndMT frequency	Acute radiation proctitis	^94^

## Effects of EndMT on cancer

EndMT is a specific mechanism resulting in CAF accumulation; antiangiogenic tumor therapy may directly decrease the abundance of activated fibroblasts that are likely to promote cancer progression^52^.

### Tumor EndMT and CAFs

ECs contribute to the abundance of CAFs through EndMT in the tumor microenvironment^52^. Using *Tie2-cre;R26Rosa-lox-Stop-lox-LacZ* transgenic mice, it was discovered that approximately 30% of fibroblastic cells (FSP^+^ cells) and 12% of α-SMA^+^ cells in the B16F10 tumor stroma were derived from EndMT (Table 1)^52^. Cancer cells can induce EndMT via TGF-β through several mechanisms. In hepatocellular carcinoma, miR-302c inhibits tumor growth through metadherin, a factor that contributes to cell motility (Table 2)^4,53^. The levels of miR-302c expressed by ECs isolated from tumor tissues were significantly lower than the corresponding levels in normal liver tissues^53^. The levels of miR-302c in ECs correlated negatively with the proliferation rate of the hepatocellular carcinoma cell line HCCLM3^53^.

Tumor-induced EndMT is mediated by factors secreted from tumor cells, such as TGF-β2 and interleukin (IL)-1β. Tumor-driven EndMT is accompanied by the activation of proinflammatory pathways in ECs^54^. The expression of cyclooxygenase-2, intercellular adhesion molecule-1, and vascular cell adhesion molecule-1 is increased, and NF-κB is activated in EndMT-transformed ECs^3^. ECs showed phenotypic changes consistent with EndMT when cocultured with OE33 esophageal adenocarcinoma cells expressing high levels of IL-1β and TGF-β2. CAFs, which were likely a result of EndMT, were found at the invasive front of esophageal adenocarcinoma, indicating the significance of EndMT in tumor progression^54^. Notably, a remarkably large number of these EndMT-derived CAFs were located close to the invasive tumor front^3^. ECs undergoing tumor-induced EndMT express higher levels of the vascular endothelial growth factor (VEGF) gene, whereas VEGF receptor 2 (VEGFR2) was downregulated in ECs^3^. EndMT-transformed esophageal ECs may be an important source of VEGF in the tumor microenvironment, and function more in a paracrine than in an autocrine manner^54^.

Loss of Tie-1, an EC-specific receptor essential for the vascular system, induces EndMT in human ECs and pancreatic tumors. Downregulation of Tie-1 triggers EndMT by activating the Slug promoter^55^. EndMT plays an important role in cancer progression and metastasis. ECs that undergo EndMT are more invasive, as they lose expression of their endothelial markers (CD31, von Willebrand factor VIII, and VE cadherin) and acquire a mesenchymal phenotype and an increased migration ability. The tumor promotes a mesenchymal shift in ECs that is regulated by Smad signaling through the synergistic stimulation of TGF-β and Notch pathways in breast cancer cells. Tumor cells increase the mesenchymal phenotypes of ECs, but maintain their endothelial phenotypes. It was shown that tumor-stimulated processes that increase extracellular matrix formation are also regulated by activation of the Notch pathway via phosphorylation of TGF-β/Smad1/5^56–60^. HSPB1 has been described as a key regulator of EndMT in lung cancer. Endothelial HSPB1 deficiency in the mesenchymal transition of vascular ECs contributes to lung fibrosis and tumorigenesis^61^.

Osteopontin is a multifunctional phospho-glycoprotein that stimulates angiogenesis in ECs. In colorectal cancer, the presence of osteopontin–integrin αVβ3 induces HIF-1α expression via a PI3K/Akt/tuberous sclerosis complex 2-mediated and mTORC1-dependent protein synthesis pathway, which transactivates TCF12 gene expression. These findings indicate that HIF-1α promotes EndMT by inducing TCF12^62^. EndMT reversal contributes to the control of chemoresistance, irrespective of the level of soluble TGF-β that is present. In a xenograft mouse model of multicellular tumor spheroids containing lung cancer cells and human umbilical vein endothelial cells (HUVECs), GSK-3β inhibition reduced the lung cancer volume by inhibiting EndMT, and had a synergistic anticancer effect on non-small-cell lung cancer cells in combination with gefitinib^63^.

The PLEK2–SHIP2 axis promotes both lung cancer cell migration (via TGF-β/PI3K/AKT signaling) and vascular invasion. This suggests that PLEK2 can serve as a valuable prognostic marker and a promising target in non-small-cell lung cancer therapy^64^. Researching functional genes and regulatory mechanisms associated with EndMT is important for revealing the mechanisms driving lung cancer metastasis and improving targeted diagnoses or treatments.

### EndMT and the tumor vasculature

EndMT also plays a major role in the tumor vasculature. ECs undergoing tumor-mediated EndMT show functional alterations, such as greater migratory capacity, higher proliferation rates, and a gain of contractile capacity, whereas the cells lose their angiogenic ability to form capillary-like tubes^54^. In glioblastoma, c-Met-mediated EC plasticity induces mesenchymal transformation to promote EC proliferation and migration, resulting in aberrant vasculature formation and chemoresistance to multiple therapies (Table 1)^19^. EndMT contributes to metastatic extravasation and intravasation. Primary tumor vasculature showing EndMT exhibits the loss of endothelial cell–cell junctions, which causes the transendothelial migration of metastatic cells^65^. For example, Rock was shown to reduce microvascular–endothelial hyperpermeability via the Rho/ROCK/MLC pathway of actin stress-fiber formation^66^. The Rho/RACK pathway rescued tight-junction integrity in brain ECs^67^. ROCK inhibition prevented tumor endothelial migration^68^. Downregulated ERG and FL1 expression caused EndMT in intratumoral ECs in B16F10 melanoma tumors, indicating the aberrant behavior of ECs in pathological environments^69^.

## EndMT in anticancer therapy

### EndMT and CAF chemoresistance

After chemotherapy, the activated stromal compartment contributes to the survival of residual cancer cells and tumor resistance^70^. CAFs promote tumor-supportive functions through cytokine and metabolite release. The CD10- and GPR77-positive CAF subset exhibits chemoresistance and poor survival in patients with breast or lung cancer^71^. CAFs may originate from ECs via EndMT, bone marrow-derived cells, adipocytes, and stellate cells^72^. Various reports have shown that CAFs mediate tumor progression and resistance, as well as relapse after chemotherapy^14^.

### EndMT and anti-VEGF therapy

Aberrant vascularization is a characteristic of resistance to chemotherapy. Human glioblastoma multiforme (GBM) tissues show an aberrant vasculature altered by EndMT. In GBM, chemoresistance and cancer progression are related to c-Met-mediated EndMT. EC-specific Met deletion resulted in EndMT, inhibited aberrant vascularization and tumor growth, and prolonged the survival of GBM-bearing mice after temozolomide treatment^19^. In GBM mouse models, PDGF-mediated EndMT decreased VEGFR2 expression via the PDGF/NF-κB/Snail axis in ECs, and thus induced resistance to anti-VEGF/VEGFR therapy. EC-specific PDGFR-B deletion sensitized tumors to anti-VEGF therapy^73^. SMA-positive perivascular cells were evaluated for their resistance to antiangiogenic therapy in vivo in malignant melanoma^74^ and pancreatic neuroendocrine tumor mouse models using RIP1-Tag2 mice^75^. Aberrant α-SMA^+^ perivascular cells were related to increased metastasis in RIP1-Tag2 mice deficient in neural cell adhesion molecule^76^. Endoglin-deficient tumors showed increased α-SMA- and NG2-positive cells in the blood vessels, and endoglin-deficient tumor ECs in RIP1-Tag2 mice caused EndMT, which induced Twist expression. Endoglin-deficient tumors exhibited more hepatic metastases than wild-type tumors, but maintained their sensitivity to anti-VEGF therapy, suggesting that the synergistic interaction of endoglin deficiency and VEGF inhibition can be used as a combined therapeutic strategy (Table 1)^77^. EC-specific miR-302c inhibited metadherin expression, reduced EndMT, and suppressed tumor growth in hepatocellular carcinoma. Metadherin and Mir-302c may be useful as antiangiogenic therapies in hepatocellular carcinoma (Table 2)^53^.

### Cardiotoxicity of anticancer drugs

Chemotherapeutic agents, including anthracycline and doxorubicin (Dox), and targeted therapies in anticancer therapy are well known to cause cardiac toxicity. Recently, data from several studies demonstrated that EndMT occurs during the development of cardiac toxicity due to anticancer chemotherapies. A recent study showed that Dox and Herceptin increased drug permeability by affecting tight-junction formation by human cardiac microvascular–endothelial cells, showing decreased expression of ZO-1 and tight-junction protein-1, which resulted in cardiotoxicity^23,78^. Cardiotoxicity is also caused by monotherapy with Herceptin (trastuzumab), a humanized monoclonal antibody used to treat breast cancers.

Calcitriol, an active form of vitamin D3 that inhibits the growth of cancer cells, has been shown to attenuate Dox-induced myocardial fibrosis and fibrotic proteins, and improve diastolic function by reducing TGF-β–Smad2-mediated EndMT and fibroblast-to-myofibroblast transition. The hearts of mice with EC-specific GFP expression showed increased levels of vimentin^+^ GFP^+^ ECs (indicative of EndMT), whereas calcitriol treatment attenuated these effects^79^. Administration of arsenic trioxide induced cardiac fibrosis in rats. In human aortic ECs, the AKT/GSK-3β/cochlear pathway was activated in arsenic trioxide-mediated EndMT, and EndMT was inhibited by the AKT inhibitor LY294002^80^.

## EndMT in radiation therapy

Approximately half of all patients with cancer are treated with radiation therapy. Despite recent technological advances, tumor radioresistance and normal tissue damage remain challenges to improving cancer-cure rates. Normal tissue damage in radiation therapy remains a dose-limiting factor^81^.

In previous studies, we reported the occurrence of EndMT in irradiated tumor and normal tissues after radiation therapy^18,61,82,83^. Radiation-induced EndMT causes fibrotic changes in both tumors and the normal tissue microenvironment, such as in the lungs and heart vessels, which contribute to tumor radioresistance and normal tissue damage, respectively. Therefore, strategies targeting EndMT may enhance the efficacy of radiotherapy to overcome normal tissue damage and tumor radioresistance.

### Radiation-induced tumor EndMT

**Fig. 1:**
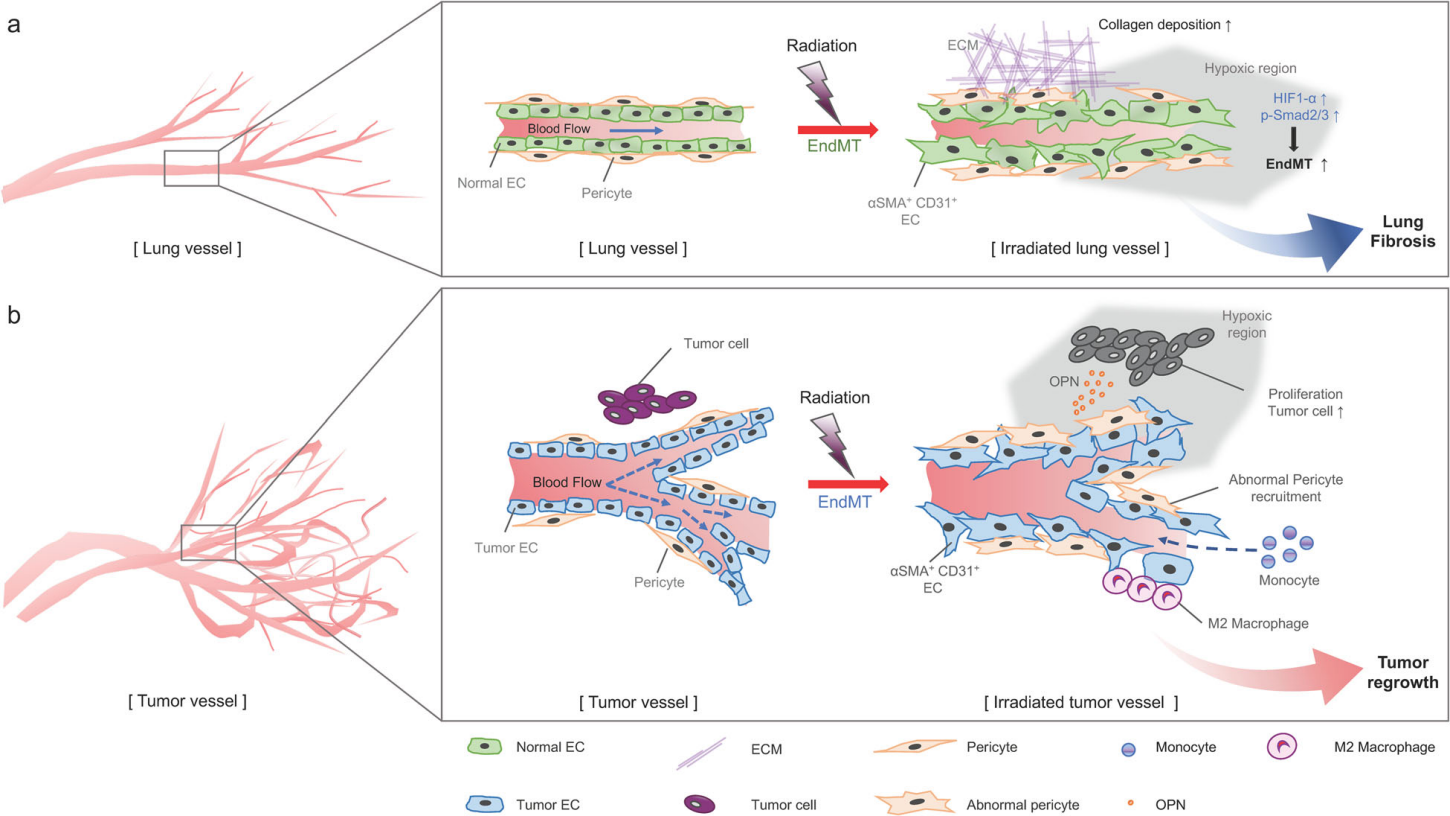
Schematic illustration of the effects of tumor EndMT on the tumor microenvironment. Mesenchymal transition of tumor ECs results in CAF formation and an abnormal tumor vasculature. **a** CAFs can affect tumor resistance to chemotherapy. **b** Fibrotic changes in ECs cause the loss of EC-specific characteristics with downregulated VEGF receptor expression, which can cause resistance to anti-VEGF therapy. **c** Immune cell infiltration into the tumor microenvironment after chemotherapy and radiation therapy can promote the generation of more protumor-immune cells via EndMT, resulting in a fibrotic vascular environment.

There is increasing interest in combining radiotherapy with antiangiogenic therapy or immunotherapy to further improve the effectiveness of radiotherapy (Fig. 1)^83^.

**Fig. 2:**
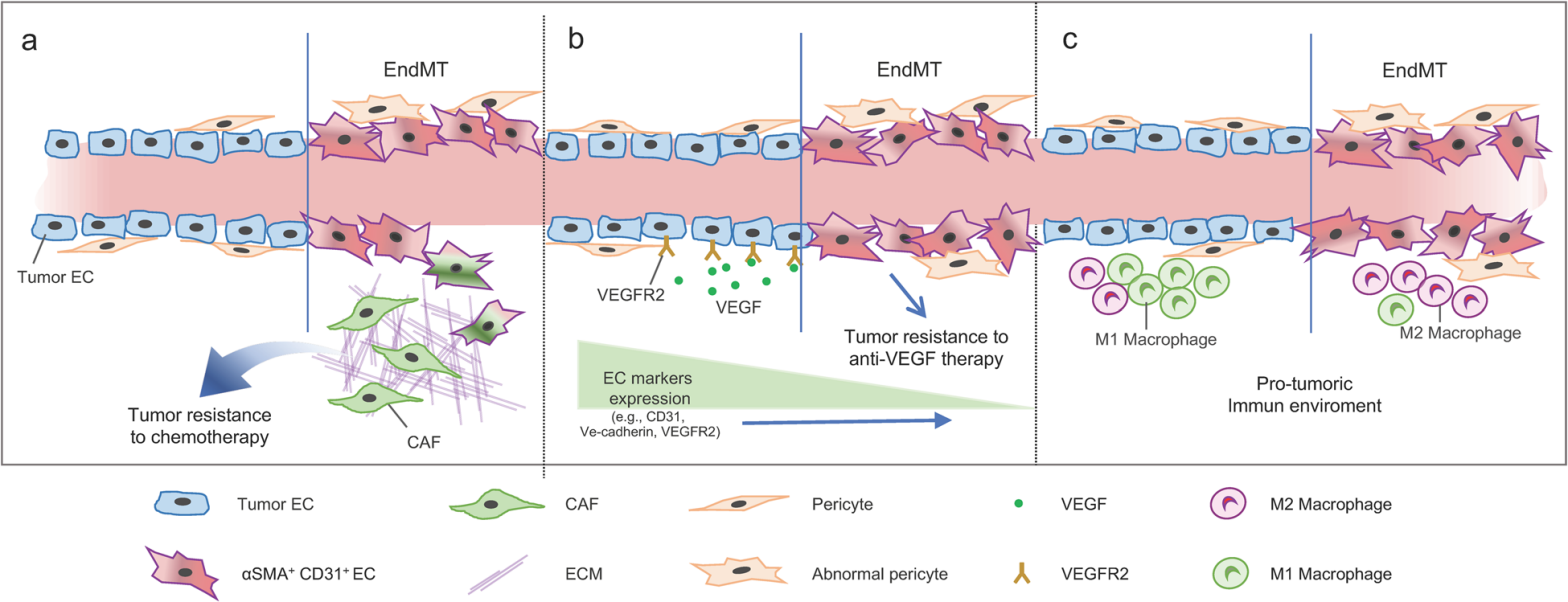
Schematic illustration of radiation-induced EndMT during radiation therapy in patients with lung cancer. **a** Radiation-induced lung vascular damage causes the mesenchymal transition of lung ECs, and thus, hypoxic regions formed by damaged vessels enhance the process of tissue fibrosis. Increased HIF-α expression on vascular ECs enhances EndMT via Smad2/3 signaling. **b** In lung cancer, radioresistant tumors can regrow after radiation therapy. ECs remaining following irradiation undergo EndMT, which increases the tumor burden, and leads to the recruitment of abnormal pericytes. Tumor EndMT promotes tumor regrowth via EndMT-related secreted molecules (such as OPN), and tumor EndMT promotes M2 macrophage polarization of monocytes recruited into the tumor microenvironment after radiation therapy.

Combining radiation therapy with antiangiogenic therapy has been reported to be effective, and VEGFR2 blockade may normalize the abnormal tumor vasculature with increased pericyte and basement membrane coverage, resulting in tumor oxygenation and an increased response to radiation therapy^84^. Therefore, understanding the tumor endothelial transition in radiation therapy may lead to more efficient combined radiotherapies. Radiation-induced EndMT results in the formation of a tumor vasculature with irregular SMA^+^NG2^+^ pericyte recruitment in adenocarcinoma lung tumors with p53 deletion and KRAS mutation (Fig. 2). EndMT has also been observed in human non-small-cell lung cancer tissues following irradiation. EC-specific p53 deletion inhibits tumor EndMT and subsequent irregular SMA^+^NG2^+^ pericyte recruitment, resulting in decreased tumor regrowth after radiation therapy (Table 1). In addition, EC-specific TGFbR2 deletion in the tumor is associated with increased tumor regrowth after radiation therapy because of enhanced TGFβR1 signaling (Table 1). Tumor EndMT occurring after radiotherapy results in osteopontin secretion, which stimulates CD44V6^+^ cancer stem cell proliferation, and contributes to the M2 polarization of macrophages. Coculture of monocytic cells and irradiated tumor ECs facilitated the conversion of monocytic cells into M2 macrophages^18^. In a neuroblastoma xenograft model, high-dose radiation therapy (HDRT) combined with Notch inhibition decreased tumor EC numbers more than HDRT alone. Notch inhibition reduced HDRT-induced EndMT, as demonstrated by decreased SMA-stained ECs. HDR increased Notch1 signaling, which was not observed at doses less than 2 Gy. These results suggest that Notch1 activation can protect tumor vessels and control the EndMT process in HDRT^85^.

### Radiation-induced vascular fibrosis

During radiation therapy, radiation-induced tissue damage can cause morbidity, which is considered a treatment-limiting factor. In radiation-induced tissue damage, vascular fibrosis is well known as a prominent occurrence^86^. Radiation directly induces DNA damage, and ionizing radiation causes ROS production and the activation of inflammatory processes, which result in fibrosis by increasing collagen deposition and reducing vascularity^87^. Blood vascular damage-induced tissue hypoxia and ischemia contribute to severe tissue injury, such as fibrosis and necrosis, and vascular fibrosis can greatly worsen the prognosis of patients undergoing radiotherapy^81,88^.

Irradiated ECs acquire a proinflammatory, procoagulant, and prothrombic phenotype, and increase the proliferation and migration of vascular smooth muscle cells^88^.

Radiation-induced vascular endothelium damage can lead to the burst release of ROS, and change the balance of angiogenesis, lipid-metabolism pathways, and immune homeostasis^89^. These acute effects can cause long-term vascular dysfunction^89^.

### Radiation-induced heart disease

Cardiovascular complications limit the use of thoracic radiation therapy for treating Hodgkin lymphoma, lung cancer, and breast cancer^90^. A meta-analysis revealed excess mortality due to cardiac disease, including cardiovascular damage, in women undergoing radiation therapy for left-sided breast cancer^91^. Atherosclerosis occurs as a delayed side effect of radiation-induced heart disease^92^.

Previously, we reported that radiation-induced EndMT occurs in human aortic ECs. Oxidized low-density lipoprotein increased radiation-induced EndMT, and irradiated ApoE^–/–^ mice showed increased oxidized low-density lipoprotein levels and a more fibrotic phenotype of ECs^82^.

### Radiation-induced intestinal damage

Gastrointestinal toxicity after radiotherapy is a major treatment-related complication. Radiation proctitis commonly occurs following inflammation of the rectal lining after treatment for cervical, prostate, and colon cancer. Recently, Mintet et al. showed that EndMT occurred during radiation proctitis development in Tie2-GFP mice, and that tissue inflammation caused the phenotypic conversion of endothelial-to-mesenchymal cells^93^. In conditional endothelial Hey2-deleted mice, EndMT occurrence and rectal tissue damage were reduced after irradiation. Microvascular protection reduced stem/clonogenic EC loss. These results suggest that EndMT can be targeted to mitigate radiation-induced intestinal damage (Table 1)^94^.

### Radiation-induced pulmonary fibrosis (RIPF)

Thoracic radiotherapy can cause RIPF as a late side effect. In particular, RIPF is one of the most frequent complications after radiotherapy for lung cancer. RIPF is characterized by fibroblast proliferation and leukocyte recruitment, as well as excessive extracellular matrix deposition. Previously, we reported that the presence of EndMT significantly increased during the early phase of RIPF development. In that study, it was demonstrated that initial vascular hypoxic damage induced EndMT at various irradiation doses in different tissue volumes. Vascular EndMT prominently appeared prior to EndMT of alveolar epithelial II cells^38^.

In irradiated human pulmonary artery ECs, EndMT was dependent on HIF-1α expression via TGF-βR1/Smad signaling. Data generated in that study revealed HIF-1α-related EndMT in human RIPF tissues^95^. A recent study provided evidence that in a coculture system of human fetal lung fibroblasts (MRC-5) and irradiated HUVECs, Snail and vimentin expression was upregulated, and CD31 expression was downregulated. Radiation-induced EndMT enhances the differentiation of fibroblasts to myofibroblasts via the Snail/miR-199a-5p axis (Table 2)^95^.

## Drugs with EndMT-inhibiting effects

Recently, several drugs in clinical testing have been reported to inhibit EndMT in various animal disease models (Table 1). These drugs inhibit EndMT by targeting various signaling molecules, such as DPP-4^25,96^, Smad^97^, TGF-β^97–99^, AMPK^100^, and other proteins^28,101–106^.

Nintedanib, which has been approved for treating pulmonary fibrosis, showed anti-vascular remodeling effects in pulmonary hypertension^28^. Macitentan was approved for treating pulmonary fibrosis and inhibited TGF-β- and ET-1-mediated EndMT in ECs isolated from patients with systemic sclerosis^98^. Recent data demonstrated that diabetic complications were related to EndMT. High glucose levels can lead to EndMT, which is regulated by miRNAs (miR-200b and miR-18a-5p) (Table 2)^107–110^.

Vildagliptin, a drug used to treat diabetes, regulates DPP-4-dependent EndMT and showed anti-fibrotic effects in a mouse model of sepsis^25^. Liraglutide, linagliptin, and losartan, which have been approved for treating diabetes and its complications, inhibited EndMT in diabetic mice and diabetic complications in mouse models^96,97,100^. In addition, the GSK inhibitor, CHIR99021, was shown to inhibit radiation-induced EndMT in HUVECs^101^. Imatinib, a PDGF receptor antagonist, regulated EndMT in pulmonary artery remodeling of pulmonary artery hypertension in rats^111^. Table 3 shows various drugs that are used clinically to inhibit EndMT.

**Table 3:** Drugs regulating EndMT in various animal disease models.

Drug	Target(s)^a^	Mechanism(s)^b^	Biological effects in EndMT^c^	Research animal model/cells^d^	Biological effect(s) (clinical phase)^e^	Clinical trial status (disease)	Refs.
Nintedanib	PDGFR, FGFR, and VEGFR	Inhibits kinase signaling pathways and EndMT	Anti-vascular remodeling effects	Pulmonary arterial hypertension	Inhibits kinases and cell proliferation	Phase 3 completed (fibrosis)	^28^
Vildagliptin	Dipeptidyl peptidase 4 (DPP-4)	Inhibits DPP-4 signaling and EndMT	Anti-fibrotic effect	Sepsis models	Blood glucose regulation	Phase 4 completed (diabetes)	^25^
Liraglutide	Glucagon-like peptide 1 (GLP-1) receptor	Inhibits high glucose-induced EndMT via the AMPK pathway	Inhibits neointima formation to increase reendothelialization	Endovascular injury in diabetic mice	Blood glucose regulation	Phase 2 completed (type-1 and -2 diabetes mellitus)	^100^
Cinacalcet	Extracellular calcium-sensing receptor	Inhibits the expression of extracellular matrix elements (type I collage and fibronectin) and EndMT	Ameliorates cardiac fibrosis	Uremic hearts	Acts as a calcium mimetic	Phase 4 completed (chronic kidney disease)	^103^
Linagliptin	DPP-4	Suppresses DPP-4 and inhibits EndMT via microRNA29 induction	Ameliorates kidney fibrosis	Streptozotocin-induced diabetic mice	Blood glucose regulation	Phase 2 completed (type-2 diabetes mellitus)	^96^
Macitentan	Endothelin-1 receptor, and endothelin B receptor	Inhibits the TGF-β- and ET-1-mediated EndMT	Inhibits fibroblast accumulation	ECs isolated from patients with systemic sclerosis (in vitro)	Antagonist of endothelin receptors on blood vessels and smooth muscle	Phase 2 completed (pulmonary hypertension)	^98^
Rapamycin (sirolimus)	Inhibited cell migration and extracellular matrix degradation	Inhibits EndMT and MMP-2/9 secretion	Inhibits EC angiogenesis in vitro	EA.hy926 cell line, a permanent endothelial cell line derived from HUVECs (in vitro)	Immunosuppressive macrolide	Phase 4 completed (transplantation, kidney)	^104^
Rapamycin	Inhibits VEGF, TGF-β, and TNF-α levels	Decreases PD-induced angiogenesis and EndMT	Protective effects, i.e., preservation of the peritoneal membrane	In vivo mouse model of peritoneal dialysis	Immunosuppressive macrolide	Phase 4 completed (transplantation, kidney)	^105^
Spironolactone	Abrogates TGF-β-induced fibrosis in EndMT	Inhibits EndMT by blocking Notch signaling and TGF-β	-	HUVECs (in vitro)	Aldosterone receptor blocker	Phase 2 completed (congenital heart disease, endomyocardial fibrosis, and heart failure)	^99^
CHIR‐99021	Reduces FSP1 and α-SMA expression	Inhibits radiation- induced EndMT	-	HUVECs (in vitro)	GSK-3 inhibitor	N/A	^101^
Losartan	Inhibits ERK phosphorylation	Inhibits EndMT	-	Hypertension, cardiac valve endothelial cells (in vitro)	Type-1 angiotensin-II receptor antagonist	Phase 2 completed (fibrosis, inflammatory reaction)	^102^
Losartan	TGF-β1/Smad2/3 pathway	Inhibits EndMT, oxidative stress damage, and the TGF-b1/Smad signaling pathway	Reduces high-fat diet-induced hyperglycemia	Diabetic nephropathy (DN)-induced renal fibrosis (in vivo)	Type-1 angiotensin-II receptor antagonist	Hypertension in the left ventricle, DN	^97^
Hydrocortisone	EPAC–RAP1 pathway	Inhibits various signaling pathways, including RAP1 activity	Enhances the barrier properties of human brain microvascular endothelial cells	Blood–brain barrier models (in vitro)	Blocks the immunosuppressive hormone cortisol	Phase 4 completed (cardiovascular insufficiency, leukemia)	^106^

^a–c^Targets, mechanism, and biological effects of drugs that were found to inhibit EndMT in vivo or in vitro.

^d^In vivo disease model used to study EndMT.

^e^Biological effects of drugs in clinical trials.

^-^ Blank: not described.

N/A not applicable.

## Concluding remarks

Taken together, the results of various studies have demonstrated that EndMT contributes to tissue fibrosis and cancer. EndMT can affect tumor progression via the mesenchymal transition of cells to CAFs, or effects on the abnormal tumor vasculature. Moreover, fibrotic changes and specific gene loss in tumor ECs may cause tumor resistance to and relapse after anti-VEGF therapy. Considering these tumor vasculature EndMT-related phenotypic changes, antiangiogenic therapies can be developed, particularly anti-VEGF therapy combined with chemo- and radiation therapy. The role of EndMT in regulating normal tissue damage, such as tissue fibrosis, should also be evaluated to reduce the side effects of anticancer therapy, such as cardiovascular damage, cardiotoxicity, and kidney and lung fibrosis. In particular, EndMT modulation may provide a new strategy for radiation therapy. Regulating EndMT in irradiated normal tissues around the tumor can reduce vascular damage and tissue fibrosis. Simultaneously, regulating tumor EndMT after radiation therapy may prevent tumor regrowth and tumor microenvironmental changes, such as those mediating the protumorigenic activities of immune cells in the fibrotic phase. Further studies are necessary to understand the exact mechanism behind tumor EndMT in human cancer.

Understanding EndMT may provide new therapeutic strategies against cancer, and reduce the related side effects, such as tissue damage.
